# Mechanical Pain Thresholds and the Rubber Hand Illusion

**DOI:** 10.3389/fpsyg.2018.00712

**Published:** 2018-05-15

**Authors:** Anna Bauer, Julia Hagenburger, Tina Plank, Volker Busch, Mark W. Greenlee

**Affiliations:** ^1^Institute of Psychology, University of Regensburg, Regensburg, Germany; ^2^Department of Psychiatry and Psychotherapy, University of Regensburg, Regensburg, Germany

**Keywords:** rubber hand illusion, body ownership, mechanical pain threshold, multisensory integration of bodily signals, proprioceptive drift

## Abstract

We manipulated the sense of body ownership with the rubber hand illusion (RHI) to determine if perception of a potentially painful threat to the rubber hand can modify the mechanical pain threshold (MPT). Simultaneous tactile stimulation of the subject’s concealed hand and the appropriately positioned visible rubber hand generated the illusion of false body ownership. The MPT was recorded on the left hand of the subjects before and after induction of the RHI, as well as during the phase in which the model hand was pricked with a sharp knife or touched by the blunt knife handle. The results indicate that the RHI could be successfully generated with our set-up. Mechanical stimuli were perceived as more painful in the condition where the rubber hand was simultaneously pricked with a knife. Our findings suggest that the illusion of body ownership gates nociceptive processing of potentially painful stimuli.

## Introduction

Our everyday perception of our world is multisensory in nature. An example in the somatosensory domain is the well-known rubber hand illusion (RHI). An appropriately positioned and visible rubber hand (RH) is simultaneously stroked with a brush while the concealed hand of the participant is stimulated with a congruent tactile stimulus. After induction of the RHI, the participant usually experiences the subjective illusion of “ownership” of the RH and usually a “proprioceptive drift” can be measured, a misrepresentation of the position of the subject’s own hand. The RHI is a striking example of how vision, touch and proprioception interact to determine our perception of our own body parts (Botvinick and Cohen, 1998).

In the present study we were especially interested in the interaction between the RHI and the multisensory aspects of pain perception. To this end, before and after the induction of the RHI, we measured mechanical pain thresholds (MPTs), while the RH was pricked with a sharp knife, as well as in three additional control conditions (see below).

Nociception is known to be modulated by multisensory input (see Höfle et al., 2010, or Senkowski et al., 2014, for a review). For example, Pomper et al. (2013) showed that spatiotemporally aligned, task-irrelevant visual stimuli enhanced the perception and processing of simultaneously induced pain in a manner as predicted by the known principle of inverse effectiveness in multisensory processing. The presence of spatially aligned low or high contrast Gabor patches enhanced pain ratings, and this effect was most pronounced in the condition with low intensity painful stimuli.

One important aspect in the context of multisensory processing of pain is also, how “ownership” of one’s own body or body parts or illusory ownership, like that induced in the RHI, influences pain perception. For example, Pia et al. (2013) investigated the interaction between pain perception and body awareness in a group of patients with lesions in the right hemisphere of the brain, who experienced the delusion that their own arm as well as the arm of an experimenter next to them belonged to their body. They also reacted with enhanced pain ratings when the experimenter’s arm experienced nociceptive stimulation. For a more general description of the central representation of pain see Craig (2003). In a non-pathological sample, Longo et al. (2009) could show that viewing one’s own body (instead of a neutral object or another person’s body part) while pain was induced with an infrared-laser, led to decreased ratings of experienced pain, thereby indicating the presence of a clear analgesic effect. This effect appeared regardless of whether the hand that was seen and perceived as one’s own was indeed stimulated by a potentially painful laser light (informative condition) or was not stimulated (un-informative condition). On the other hand, Torta et al. (2015) found in an ERP study that vision of the body affected nociceptive and non-nociceptive processing differently, but did not find a significant effect of vision on the perceived pain intensity. In a study by Höfle et al. (2012) the authors presented video clips to their participants allegedly showing their own hands either touched by a cotton swab (non-painful condition) or pricked by a needle (painful condition) while their real hand was stimulated electrically in a painful or non-painful manner. The participants should rate intensity and pleasantness of the sensation. Here, seeing a needle prick clearly increased unpleasantness ratings in comparison to seeing the cotton swab touch. In a new pilot randomized control trial, Mithal et al. (2018) tested participants, who were instructed to either look at the needle or to look away from the needle during vaccination. While the self-reported sensation of fear was higher in the group who was told to look at the needle, no difference was found in the self-reported sensation of pain in the two groups.

Other studies more directly investigated the connection between the RHI and the perception of pain. For example, Capelari et al. (2009) induced the RHI with tactile and tactile-painful stimuli and found that the illusion could also be produced by tactile-painful stimulation. This finding indicates that the RHI can also be induced by appropriate nociceptive stimulation. Other studies point to a possible connection between the RHI and thermal pain threshold changes. Some investigators found decreased temperature sensitivity (Llobera et al., 2013), reduced discomfort to cold (Siedlecka et al., 2014), increased pain thresholds (Martini et al., 2014) or increased pain tolerance in a cold pressor ice bath (Giummarra et al., 2015) on the concealed hand after induction of the RHI. In contrast, Mohan et al. (2012) found no pain relief with the RHI, also applying thermal stimuli. Valenzuela-Moguillansky et al. (2011) measured pain ratings in response to thermal stimuli in two RHI experiments. In their first experiment, they found a decrease of pain ratings in comparison to a non-stroking control condition, where the RH was only viewed, while tactile stimulation was applied to the real hand. In comparison to the findings of an asynchronous control condition (their experiment 2), a relative increase in pain ratings was found after induction of the RHI. They discuss their conflicting findings in the context of different degrees of body ownership or disownership. In another study, not focusing on thermal stimuli, Armel and Ramachandran (2003) could show that, following induction of the RHI, subjects expressed distress when one finger of the RH was bent into a painful pose, evidenced by significant skin conductance response (SCR) on the concealed, true hand, which was not injured.

The aim of our study was to extend previous results by using mechanical stimuli. We investigated whether MPTs could be altered by inducing the RHI. The MPT is assumed to be closer to clinical pain than thresholds measured with thermal stimuli. In our main experiment, we measured the MPT while the RH was pricked with a sharp knife. We expected that the thresholds would decrease when the subjects viewed the RH while it was being subjected to a potentially painful stimulus (a knife prick) simultaneously with the measurement of the pain threshold in the real hand. In three control experiments we additionally investigated, if effects on the MPT also occur without successful induction of the illusion (asynchronous stimulation during application of RHI, control experiment 1), without painful stimulation of the RH (touching the RH with the knife handle, control experiment 2) or without even watching the RH, while the MPT is assessed (control experiment 3).

## Materials and Methods

### Main Experiment

#### Participants

Forty-five participants (38 female and 7 male), all right-handed, were included in the main experiment (mean age: 22.4 years; *SD* = 3.9 years). None of the experimental participants reported any history of neurological or psychiatric illness, nor illnesses of the peripheral or central nervous system. All participants were informed about the procedure of the experiment and they had to sign a declaration of informed consent prior to the participation.

This study was carried out in accordance with the recommendations of local ethic committee of the University of Regensburg. The protocol was approved by the local ethic committee of the University of Regensburg. All subjects gave written informed consent in accordance with the Declaration of Helsinki.

#### Procedure

**FIGURE 1:**
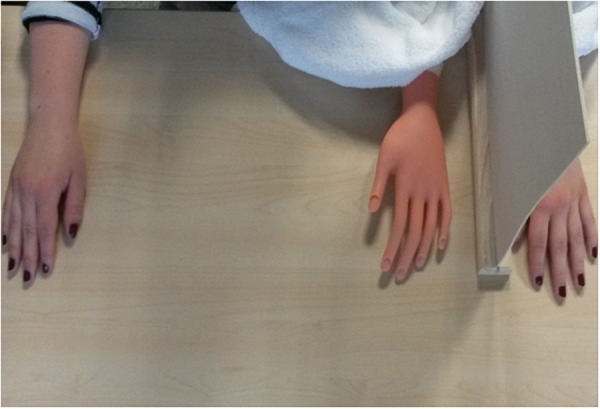
Experimental setup in the present rubber hand illusion (RHI) study. The middle finger of the rubber hand (RH) was centered on the zero position of the measurement tape fixed to the underside of the table top (not visible to the subject). The middle finger of the subject’s left hand was centered 20 cm to the left and was visually occluded by a gray wooden partition.

Most participants were tested to the same time of day (between 8 and 12 a.m.) and in the same laboratory according to a part of the standardized protocol of quantitative sensory measuring (QST) for MPT (Rolke et al., 2006b). At the beginning of the experiment the participants sat at a table and were asked to position their hands on tagged positions on the table top (see **Figure 1**). On the underside of the table, a measurement tape was attached at the forefront. Accordingly, the left middle finger was at “0 cm” and the middle finger of the RH lay at “20 cm”. Proprioceptive drift could be thus determined in terms of positions along the measurement tape. The illusion strength was also assessed by Botvinick and Cohen’s (1998) questionnaire. It consists of nine statements (seven-step visual analog scale) and was translated into German by author AB. The first three statements (see Supplementary Table S1) described the strength of the illusion; the other six were used as control questions.

With the help of a wooden partition (34 cm height and 30 cm width), the participant’s left hand was occluded from sight. The left arm was covered up to the shoulder with a white, opaque towel. As a consequence, the participants could not see their left hand or arm at any time during the experiment.

After the participant’s hands were positioned, the proprioceptive drift was measured. This is the distance between the indicated and the actual position of the left middle finger of the participant’s own hand. The participants were asked to close their eyes. Then they moved their right index finger along the tape on the underside of the table with the forefinger until they felt to have reached the position of the left middle finger. The experimenter noted the position of the right index finger on the measuring tape.

Immediately after this measurement, the MPT, selected from the standardized test battery QST (Rolke et al., 2006b), was measured. The measurement was carried out in accordance to the guidelines of the QST (Rolke et al., 2006a). Participants closed their eyes during the baseline measurement of the MPT. Blunt needles, called pinpricks, were used with a stimulus intensity of 8, 16, 32, 64, 128, 256, and 512 mN. We used the “method of limits” according to the QST protocol (Rolke et al., 2006b). Taken together, the subject was required to indicate as soon as an increasingly strong pin prick stimulus was detected (ascending ramp), or when a decreasing stimulus was no longer detected (descending ramp).

After this, the examiner stroked the RH and the left hand of the participants with two brushes synchronously for 2 min. The participants viewed the stroking of the RH and felt the tactile stimulation of their left hand. After induction of the RHI, the proprioceptive drift was measured again. Then the participants watched the RH being pricked visibly with a knife while the MPT was determined on their real hand. Each application of the pinprick was accompanied synchronously by the knife prick at the appropriate location on the RH. The participant had again to decide whether the stimulus was perceived as “painful” or “not painful.” Upon conclusion of these measurements, the participants were asked to complete Botvinick and Cohen’s (1998) questionnaire.

### Control Experiment 1 – Asynchronous Stimulation During Induction of RHI

#### Participants

Twenty participants (17 female and 3 male), all right-handed, were included in control experiment 1 (mean age: 21.9 years; *SD* = 4.6 years). They were all tested by author JH and were also subjects of the main experiment. The main experiment and control experiment 1 were conducted on two different days. Half of the subjects started with the main experiment, the other half with control experiment 1.

#### Procedure

The procedure was comparable to the main experiment, with the exception that now, during the phase of induction of the RHI, RH, and real hand were stimulated asynchronously by a delay of approximately 2 s with the brushes for 2 min.

Proprioceptive drift and MPTs were measured before and after the induction phase of the RHI in the same manner as described in the procedures of the main experiment. Also the RHI questionnaire (see Supplementary Table S1) was completed at the end of data collection by the participants.

### Control Experiment 2 – Rubber Hand Touched With Back of Knife Handle

#### Participants

The same 20 participants as in control experiment 1 took part in control experiment 2, again all tested by author JH. The main experiment and control experiment 2 were conducted on the same day for this subgroup of subjects, separated by a short break. On that day, half of the subjects started with the main experiment, the other half with control experiment 2.

#### Procedure

The procedure was comparable to the main experiment, with the exception that the participants now watched the RH being touched visibly with the back of the knife handle (“no pain condition”), while the MPT was determined on their real hand. Proprioceptive drift and MPTs were measured before and after the induction phase of the RHI in the same manner as described in the procedures of the main experiment. No additional RHI questionnaire was given to the participants, they only completed one questionnaire at the end of the session that contained the main experiment and control experiment 2.

### Control Experiment 3 – Eyes Closed During MPT

#### Participants

Twenty-five participants (21 female and 4 male), all right-handed, were included in control experiment 3 (mean age: 22.8 years; *SD* = 3.3 years). They were all tested by author AB and were also participants of the main experiment. This group of participants completed the main experiment and control experiment 3 on the same day, separated by a short break and starting with the control experiment.

#### Procedure

The procedure was again comparable to the main experiment, but now, after the induction of the RHI (by synchronous stroking of RH and real hand), the MPT was measured in the same manner as in the baseline measurement – with eyes closed (i.e., no visual feedback).

## Results

### Main Experiment: Pricking Rubber Hand With Knife Point

#### Proprioceptive Drift and RHI-Questionnaire

**FIGURE 2:**
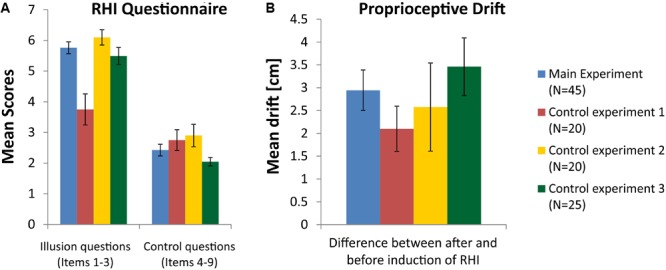
**(A)** Mean scores for the illusion questions (items 1–3) and control questions (items 4–9) in the RHI questionnaire (see Supplementary Table S1). **(B)** Mean differences of proprioceptive drift between measurements before and after induction of the RHI. Blue columns represent data from the main experiment, red columns for control experiment 1, yellow columns for control experiment 2, and green columns for control experiment 3. The data of all 45 participants entered the analysis in the main experiment, while control experiment 1 and 2 were completed by 20 subjects and conducted by examiner JH and control experiment 3 was completed by 25 subjects and conducted by examiner AB (error bars depict SE).

The analysis of the proprioceptive drift in the main experiment with *N* = 45 participants indicated that a significant shift occurred after induction of the illusion, where the subjectively estimated position of the left middle finger shifted toward the location of the RH. The *t*-test for paired comparisons yielded a significant shift increase after the induction of the illusion [*t*(44) = 6.64; *p* < 0.001]. An evaluation of the questionnaire data revealed a significant change in response to the three illusion questions compared to the six control questions [*t*(44) = 12.62; *p* < 0.001], indicating that induction of the illusion was successful. **Figure 2** (blue columns) shows the mean values of questionnaire scores (**Figure 2A**) and drift differences (**Figure 2B**) in the main experiment.

#### Pain Thresholds

**FIGURE 3:**
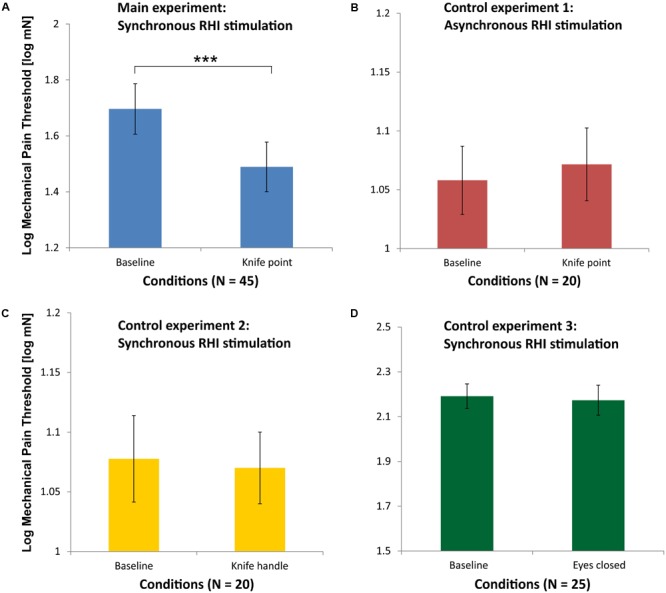
Mean Log mechanical pain thresholds (MPT) for the main experiment (blue, **A**), control experiment 1 (red, **B**), control experiment 2 (yellow, **C**), and control experiment 3 (green, **D**). Please note that the abscissae differ in scaling for sake of clarity. The data of all 45 participants entered the analysis in the main experiment, while control experiment 1 and 2 were completed by 20 subjects and conducted by examiner JH and control experiment 3 was completed by 25 subjects and conducted by examiner AB (^∗∗∗^*p* < 0.001; error bars depict SE).

Because MPTs were not normally distributed, they were logarithmically transformed for parametric statistical testing. **Figure 3A** shows the mean Log MPT for the baseline measurement of the MPT before induction of the RHI (Baseline) in comparison to the mean Log MPT after induction of the RHI, while participants saw the RH being pricked by a knife synchronously (*N* = 45). Mean Log MPT in the latter condition decreased significantly in comparison to the baseline condition as analyzed by a *t*-test for repeated measures [*t*(44) = 4.41; *p* < 0.001]. Overall, collected pain thresholds differed between the two examiners (authors AB and JH), an effect that is known from the literature (e.g., Geber et al., 2011). Therefore, we tested the conditions separately for MPTs collected by AB (*N* = 25) and MPTs collected by JH (*N* = 20). For examiner AB, mean Log MPTs also differed significantly between baseline and knife condition [*t*(24) = 4.49; *p* < 0.001]. A similar result we obtained for examiner JH [*t*(19) = 2.25; *p* = 0.036].

### Control Experiment 1: Asynchronous Stroking During Application of RHI

#### Proprioceptive Drift and RHI-Questionnaire

The analysis of the proprioceptive drift within the group of subjects, who participated in control experiment 1, also indicated that a significant shift occurred after induction of the illusion, although the stroking of RH and real hand was done asynchronously. The *t*-test for paired comparisons yielded a significant shift increase after the induction of the illusion [*t*(19) = 4.24; *p* < 0.001]. An evaluation of the questionnaire data in this group revealed no significant difference between the three illusion questions and the six control questions [*t*(19) = 1.95; *p* = 0.07], indicating that induction of the illusion was not successful or at least largely smaller than with synchronous stroking. **Figure 2** (red columns) shows the mean values of questionnaire scores and drift differences in control experiment 1. Scores for the illusion questions (items 1–3) differ significantly between the main experiment and control experiment 1 (*p* < 0.001).

#### Pain Thresholds

As can be seen in **Figure 3B**, there was no significant difference in the mean Log MPTs between the baseline condition and the knife condition [*t*(19) = 0.15; *p* = 0.88], when RH and real hand were asynchronously stroked by brushes in the RHI induction phase.

### Control Experiment 2: Touching of Rubber Hand With Back of Knife Handle

#### Proprioceptive Drift and RHI-Questionnaire

The analysis of the proprioceptive drift within the group of subjects, who participated in control experiment 2, indicated that a significant shift occurred after induction of the illusion. The *t*-test for paired comparisons yielded a significant shift after the induction of the illusion [*t*(19) = 2.67; *p* = 0.015]. Since this condition with the knife handle was also applied in the asynchronous stroking condition, a repeated measures ANOVA on the group of 20 subjects with the factors synchronicity (synchronous and asynchronous) and time point (before RHI and after RHI) was conducted. It yielded a significant main effect of time point [*F*(1,19) = 6.87; *p* = 0.017] and a marginally significant main effect of synchronicity [*F*(1,19) = 4.08; *p* = 0.058], indicating that the proprioceptive drift toward the RH tended to be larger in the condition with synchronous stroking. An evaluation of the questionnaire data in this group of subjects, who participated in control experiment 2, revealed a significant increase in response to the three illusion questions compared to the six control questions [*t*(19) = 6.53; *p* < 0.001)], indicating that induction of the illusion was successful. **Figure 2** (yellow columns) shows the mean values of questionnaire scores and drift differences in control experiment 2.

#### Pain Thresholds

As can be seen in **Figure 3C**, there is no significant difference in the mean Log MPTs between the baseline condition and the knife condition, when the RH is touched with the knife handle [*t*(19) = 0.28; *p* = 0.78]. This condition was also applied after the asynchronous stroking phase (not shown in **Figure 3**). A repeated-measures ANOVA on these data of the 20 participants with the factors synchronicity (synchronous and asynchronous) and knife condition (baseline and knife handle) yielded no significant main effects or interaction (all *p* > 0.1).

### Control Experiment 3: Eyes Closed During MPT

#### Proprioceptive Drift and RHI-Questionnaire

The analysis of the proprioceptive drift within the group of subjects, who participated in control experiment 3, indicated that a significant shift occurred after induction of the illusion, where the subjectively estimated position of the left middle finger shifted toward the location of the RH. The *t*-test for paired comparisons yielded a significant shift increase after the induction of the illusion [*t*(24) = 5.47; *p* < 0.001]. An evaluation of the questionnaire data in this group revealed a significant change in response to the three illusion questions compared to the six control questions [*t*(24) = 12.37; *p* < 0.001)], indicating that induction of the illusion was successful. **Figure 2** (green columns) shows the mean values of questionnaire scores and drift differences in control experiment 3.

#### Pain Thresholds

As can be seen in **Figure 3D**, there is no significant difference in the Log MPT between before (Baseline) and after the illusion (as measured with closed eyes) [*t*(24) = 0.53; *p* = 0.60].

## Discussion

The results from the main experiment show that, following induction of the RHI, pain thresholds measured on the participant’s left hand were significantly lower when they viewed the RH being pricked by a sharp knife. This effect was not observed without previous successful induction of the RHI, as shown by control experiment 1. The illusion was less striking and MPTs did not differ between baseline measurement before RHI induction and the measurement during the knife prick after RHI induction, if RH and own hand were stroked asynchronously during the RHI induction phase. Interestingly, a shift of the perceived position of the real hand toward the RH, as measured by proprioceptive drift, occurred in both cases, main experiment and control experiment 1, but the shift was less pronounced in control experiment 1. Furthermore, subjective ratings of ownership, as measured by questionnaire, point to an overall less vivid or absent illusion of ownership of the RH in control experiment 1.

Likewise, the view of a non-threatening, non-painful stimulus on the RH (control experiment 2, RH touched by the knife handle) did not alter MPTs. Our results are in line with Höfle et al. (2012), who also found that watching a needle prick on a hand perceived as the participants’ own hand increased unpleasantness ratings of electrical stimuli more than watching the hand touched by a non-painful Q-tip. Höfle et al. (2012) discuss their findings in the context of expectation driven by previous experience. Similarly, in our study autobiographical experience suggested to the participants that contact with the knife point should hurt more than contact with the knife handle. The results stand in contrast to those of Longo et al. (2009), who found analgesic effects on participants’ pain perception by watching a hand perceived as being their own during application of painful stimuli. But the difference might be explained by the different stimulus material used. While Longo et al. (2009) presented a laser light that – potentially – did not visibly injure or damage the hand that was seen (therefore possibly leading to reduced ratings of pain intensities or unpleasantness), the needle prick seen in the video clips presented by Höfle et al. (2012) more obviously hurt the hand that was seen. Similarly, the prick with the sharp knife in our experiment visibly “hurt” the RH, while the touch with the knife handle did not. A similar experiment to the one of Höfle et al. (2012) was performed by Valeriani et al. (2008), where subjects saw video clips of the hands of others pricked by a needle or touched by a cotton swab, while the subjects themselves received painful stimuli at their own corresponding hand induced by a laser. In those experiments, the video clips and the painful stimulation on the subjects’ own hands were obviously not synchronized (Höfle et al., 2010), so that – similarly to our asynchronous RHI condition (control experiment 1) – “ownership” of the hand seen in the video clip could not fully occur. Accordingly, no effects of visual input on pain intensity and unpleasantness ratings were observed by Valeriani et al. (2008).

Our findings are further in line with those of Kanaya et al. (2012), who showed that temperature sensation in the real (hidden) hand were affected by the RH being brought in contact with hot or cold objects. Also, Giummarra et al. (2015) found hyperalgesia, when the RHI was induced on a “wounded” RH. Thus, in our study, the viewing of the knife pricking the finger of the RH and feeling the blunt needle on the hidden hand appear to have influenced the pain perception on the real hand. The effect observed in the main experiment suggests the idea that the RH has been successfully “incorporated” into the participants’ body percept (see also Valenzuela-Moguillansky et al., 2011). Potentially painful threats to the RH led to alterations in pain sensitivity in the real, but hidden from view, hand.

We observed no significant alteration for the MPTs without watching the RH (see control experiment 3). Hence the induction of the RHI alone did not change the MPT values significantly. Thus, we could not find any pain relief due to inducing the RHI, when measuring the MPT, similar to Mohan et al. (2012), but in contrast to Martini et al. (2014), who both used thermal stimuli. In the context of different degrees of body awareness or ownership, as discussed by Valenzuela-Moguillansky et al. (2011), a “disownership” of the own real hand appears not to have taken place. Changes in the cortical representations of the contralateral upper limb in the insular cortex could be a potential neural correlate of altered body ownership (Craig, 2009).

In summary, the MPT seemed to remain relatively stable during the induction of the RHI. Nevertheless, apparently painful stimulation of the RH actually resulted in a decrease of the pain thresholds in the real hand. These results suggest that this feigned injury was interpreted by the brain as real pain. As a consequence, the pain thresholds to pinpricks on the real hand decreased. Pain thresholds for mechanical stimuli (here: MPT) appear to be robust in the presence of the illusion, but they altered by a feigned threat to the RH.

Our data enlarges our knowledge about the modulation of pain perception by the sense of body ownership. As such our findings may provide further insight into related phenomena like that of the phantom-limb pain experienced by amputees (e.g., Ramachandran et al., 1998). Once body ownership is established, any threat of noxious stimulation to the new surrogate limb induces transient hyperalgesia in the corresponding (albeit hidden) limb.

## Author Contributions

AB, JH, TP, MG, and VB developed and designed the study. AB and JH performed the experiment. AB, JH, and TP analyzed the data. AB, TP, VB, and MG wrote the main manuscript text. AB and TP prepared the figures. All authors reviewed the final manuscript.

## Conflict of Interest Statement

The authors declare that the research was conducted in the absence of any commercial or financial relationships that could be construed as a potential conflict of interest.
