# Supplementary material for: Mechanical Pain Thresholds and the Rubber Hand Illusion
Source: Front Psychol. 2018 May 15;9:712. doi: 10.3389/fpsyg.2018.00712 (PMC5962648; doi:10.3389/fpsyg.2018.00712)
Supplement: Supplementary file 1 [file Table_1.docx]

**Supplementary Table S1:**

Table S1: Questionnaire items in their original version and the translated version (German)

| Original statements  (Botvinick & Cohen, 1998) | Translated Version |
| --- | --- |
| 1. It seemed as if I were feeling the touch of the paintbrush in the location where I saw the rubber hand touched. | 1. Es schien so, als ob ich die Berührung mit dem Pinsel an der Stelle spürte, an der ich auch die Berührung an der Gummihand sah. |
| 1. It seemed as though the touch I felt was caused by the paintbrush touching the rubber hand. | 1. Es schien so, als ob die Berührung, die ich fühlte, vom Pinsel erzeugt wurde, der die Gummihand berührte. |
| 1. I felt as if the rubber hand were my hand. | 1. Es fühlte sich so an, als ob die Gummihand meine Hand wäre. |
| 1. It felt as if my (real) hand were drifting towards the right (towards the rubber hand). | 1. Es fühlte sich so an als ob sich meine (echte) Hand zur Gummihand hinbewegen würde. |
| 1. It seemed as if I might have more than one left hand or arm. | 1. Es schien so, als ob ich mehr als eine linke Hand/ einen linken Arm hätte. |
| 1. It seemed as if the touch I was feeling came from somewhere between my own hand and the rubber hand. | 1. Es schien so als würde die Berührung, die ich fühlte, von irgendwo zwischen meiner eigenen und der Rubber Hand kommen. |
| 1. It felt as if my (real) hand were turning ‘rubbery’. | 1. Es fühlte sich so an, als ob meine Hand „gummiartig“ werden würde. |
| 1. It appeared (visually) as if the rubber hand were drifting towards the left (towards my hand). | 1. Es erschien (visuell) so, als ob sich die Gummihand nach links (in Richtung meiner eigenen Hand) bewegen würde. |
| 1. The rubber hand began to resemble my own (real) hand, in terms of shape, skin tone, freckles or some other visual feature. | 1. Die Gummihand begann meiner eigenen (echten) Hand hinsichtlich der Form, der Hautfarbe, der Sommersprossen und anderer visueller Eigenschaften zu ähneln. |
